# Emerging Roles of LncRNAs in the EZH2-regulated Oncogenic Network

**DOI:** 10.7150/ijbs.63488

**Published:** 2021-07-25

**Authors:** Aixin Hao, Yunxuan Wang, Daniel B. Stovall, Yu Wang, Guangchao Sui

**Affiliations:** 1 Key Laboratory of Saline-alkali Vegetation Ecology Restoration, Ministry of Education, College of Life Science, Northeast Forestry University, Harbin 150040, China; 2 Department of Medical Oncology, Harbin Medical University Cancer Hospital, Harbin, 150081, China; 3 College of Arts and Sciences, Winthrop University, Rock Hill, SC 29733, the United States

**Keywords:** cancer, lncRNA, EZH2, PRC2, H3K27me3, non-histone methylation, epigenetic regulation

## Abstract

Cancer is a life-threatening disease, but cancer therapies based on epigenetic mechanisms have made great progress. Enhancer of zeste homolog 2 (EZH2) is the key catalytic component of Polycomb repressive complex 2 (PRC2) that mediates the tri-methylation of lysine 27 on histone 3 (H3K27me3), a well-recognized marker of transcriptional repression. Mounting evidence indicates that EZH2 is elevated in various cancers and associates with poor prognosis. In addition, many studies revealed that EZH2 is also involved in transcriptional repression dependent or independent of PRC2. Meanwhile, long non-coding RNAs (lncRNAs) have been reported to regulate numerous and diverse signaling pathways in oncogenesis. In this review, we firstly discuss functional interactions between EZH2 and lncRNAs that determine PRC2-dependent and -independent roles of EZH2. Secondly, we summarize the lncRNAs regulating EZH2 expression at transcription, post-transcription and post-translation levels. Thirdly, we review several oncogenic pathways cooperatively regulated by lncRNAs and EZH2, including the Wnt/β-catenin and p53 pathways. In conclusion, lncRNAs play a key role in the EZH2-regulated oncogenic network with many fertile directions to be explored.

## Introduction

Cancers are characterized by dysregulated genetic and epigenetic homeostasis under environmental and endogenous stimuli, leading to uncontrolled cell division, growth and life-threatening metastasis 1. Malignant transformations are generally caused by accumulated genetic mutations of oncogenes and tumor suppressors involved in various important signaling pathways, but mechanisms underlying cancer development and progression can be extended to the level of epigenetic regulation 2, 3. Epigenetics is defined as regulatory mechanisms leading to stably inheritable changes resulting from alterations of gene expression without alterations in DNA sequences 4, 5. In the past decades, cancer epigenetics has gained considerable attention due to our increasing understanding of its regulation in cell fate decisions, reversible and environment-dependent features, and vulnerability as therapeutic targets 6-8. DNA methylation and histone modification are two important epigenetic mechanisms regulating gene expression. Meanwhile, noncoding RNAs (ncRNAs), a large population of molecules with currently undetermined numbers, are closely involved in different epigenetic processes, either directly or indirectly, to modulate gene expression, RNA splicing and stability, chromatin remodeling, and genomic stability 9, 10.

With the development of high-throughput technologies and computational algorithms, we have learned that only about 2% of the human genome codes proteins, and most transcripts from over 70% of the genome are ncRNAs 11-13. In the past decade, ncRNAs have been intensively studied in the context of their regulatory roles in gene expression 9, 10. Long noncoding RNAs (lncRNAs) are defined as a class of ncRNAs longer than 200 nucleotides without evident protein coding ability. LncRNAs could modulate gene expression by serving as molecular signals, decoys, scaffolds and guides through binding both nucleic acids and proteins 14, 15. Based on these features, emerging evidence demonstrates that lncRNAs are involved in almost all progressive stages of cancers, including cell migration, invasion, metastasis and angiogenesis, as well as autophagy 16-18. Since the expression of lncRNAs exhibit more tissue- and cell-specific patterns than protein-coding genes, they have been considered as potential biomarkers and therapeutic targets in cancers 19. For example, a well-characterized lncRNA, HOTAIR (HOX Transcript Antisense RNA), has been recognized as a prognostic marker of several cancer types 20-22. Mechanistically, HOTAIR acts as a molecular scaffold for PRC2 (Polycomb repressive complex 2) and other histone modification complexes, such as LSD1, to establish gene silencing 23, 24.

Enhancer of zeste homolog 2 (EZH2), a well-known SET domain-containing protein, is the catalytic core of the PRC2 with methyltransferase activity 25. PRC2 is essential for transcription silencing through specifically catalyzing the tri-methylation of lysine 27 on histone H3 (H3K27me3) 26. EZH2 expression is commonly elevated in nearly all solid cancers, including gastric, breast and prostate cancers, which is associated with poor prognosis, suggesting an oncogenic role of EZH2 in these cancers 25. Noteworthily, in lymphoma and myeloid neoplasms, several EZH2 missense mutations and truncations were reported to associate with oncogenic activities 27-29. In most studies, EZH2 inhibition could reduce tumor formation and promote cell death. Recent reports also demonstrated EZH2 as a promising target to suppress tumor immune escape and block viral infection 25, 30-32. Thus, EZH2 has been considered a bona fide therapeutic target in cancer therapies, and inhibitors targeting EZH2 have been increasingly developed, such as tazemetostat (EPZ-6438), valemetostat, CPI-0209 and CPI-1205 33-36. Tazemetostat was the first drug approved by the FDA in 2020 for the treatment of patients with locally advanced or metastatic epithelioid sarcoma not eligible for complete resection 37, and phase Ⅰ/Ⅱ clinical trials are still ongoing in the treatments of other cancers, such as diffuse large B-cell lymphoma and malignant mesothelioma 33.

In addition, an increasing number of studies indicate that lncRNAs regulate the expression and function of EZH2 at multiple levels. On the other hand, many lncRNAs involved in different oncogenic signaling pathways are also transcriptional targets of EZH2. In the current review, we will discuss functional interplays between EZH2 and different lncRNAs, and the biological consequences of their interactions, mostly in the contexts relevant to oncogenesis.

## LncRNAs regulate a dual role of EZH2 in PRC2-dependent transcription

**Figure 1:**
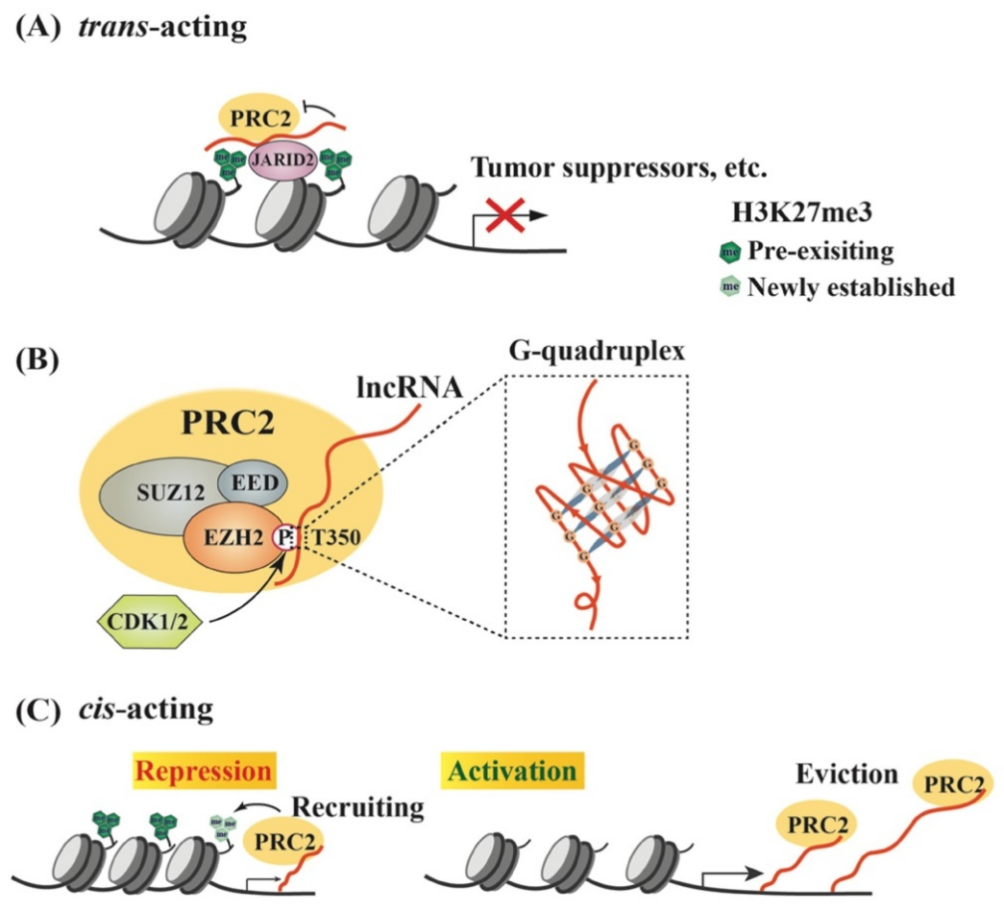
** Models of lncRNA-mediated EZH2 action to regulate chromatin remodeling.** (A) Schematic model of *trans* lncRNAs recruiting EZH2 to catalyze H3K27me3 and repress gene transcription. LncRNA (red curve, the same below) recruits PRC2 to the promoter of target genes, such as tumor suppressor genes, but inhibits the methyltransferase activity of EZH2, which can be relieved by JARID2 binding. (B) Characteristics of interaction between EZH2 and lncRNAs. EZH2, EED and SUZ12 are the core subunits of PRC2. Phosphorylation of EZH2 at T350 mediated by CDK1/2 is essential for its association with lncRNAs, and preferentially binds to G-quadruplex RNA. (C) Regulation of PRC2 activity by *cis*-acting RNAs. PRC2 scans nascent RNAs and then binds to G-tract regions. With slow transcription, EZH2 binding to nascent RNAs can promote H3K27me3 on chromatin to repress gene expression; with fast transcription, pre-existing PRC2 can bind nascent RNAs and then be evicted from the promoter, leading to gene activation.

SUZ12, EZH2 and EED comprise the core components of PRC2 that serves as an epigenetic “writer” to switch off genes 26. Although H3K27 methylation is an important event to determine gene expression status and cell fate, it remains poorly understood how it is catalyzed by PRC2 in a site-specific manner. Early studies revealed that many molecules could act as “readers” or recruiters of PRC2 to promote *de novo* H3K27me3 38-42, such as RBBP4/7, JARID2 and YY1 43-45. However, a growing number of lncRNAs have been demonstrated to serve as “genomic address codes” for PRC2 46, including HOTAIR, PVT1 and many other lncRNAs (Table 1). A potential mechanism is that lncRNAs preferentially bind a GA-rich DNA motif in promoters to form a triplex, and then recruit protein complexes, such as PRC2 47-51. As an example of these studies, the HITT is an EZH2-binding lncRNA, and its additional region forms an RNA-DNA triplex with the HIF-1α promoter, leading to PRC2 recruitment and reduced HIF-1α expression under normoxic conditions. This suppression is released and HIF-1α expression is activated under hypoxic conditions due to reduced HITT levels 51. Actually, computational tools, such as Triplexator and Triplex Domain Finder (TDF), were developed to evaluate triplex-forming lncRNAs and their potential target sites in the human genome 49, 52. In addition, another software, Triplex-Inspector, can also aid the design of sequence-specific ligands and selection of optimal targets to avoid off-target effects in genomic manipulations 53. The PRC2- or EZH2-associated lncRNAs exert critical regulatory activities in the expression of various genes involved in different oncogenic signaling pathways. Interestingly, many of these lncRNAs inhibit the methyltransferase activity of EZH2 unless PRC2 associates with JARID2, which reduces PRC2-RNA interaction and subsequently promotes EZH2 activity 54-56 (Figure 1A).

To date, a large number of lncRNAs have been identified to interact with PRC2 95, 96. Both EZH2 and EED contribute to PRC2 binding to lncRNAs, and EZH2 has much higher affinity to lncRNAs than EED, although no canonical RNA recognition motif (RRM) has been identified in either protein 55, 97. Thus, the heterodimer EZH2-EED is necessary and sufficient for HOTAIR binding 97. Phosphorylation at T350 of EZH2 (pT350) mediated by CDK1 and CDK2 is crucial for PRC2 binding to its lncRNA recruiters 98-100 (Figure 1B). Consistently, the phosphorylation of mouse Ezh2 at T345 (corresponding to T350 of human EZH2) was also important for its binding to lncRNAs HOTAIR and Xist, and the T345D mutant also showed increased affinity to the lncRNAs compared to wild type Ezh2 and T345A mutant 101. In addition, Ezh2-T345 is located in a ncRNA-binding domain (ncRBD1, amino acids 342-370), overlapping with the BRCA1 binding region (amino acids 341-559) on Ezh2 102. Therefore, BRCA1 could act as a negative regulator of PRC2 through competing for EZH2 association with lncRNAs 103.

Research efforts have been made to investigate whether any special RNA sequence or motif is favored by PRC2. In HOTAIR, a highly structured domain consisting of 89 nucleotides was identified as an EZH2 binding region 97. This G-rich region forms alternative G-quadruplex structures depending the presence of potassium ions, which is different from a reported PRC2-binding tandem dual-hairpin motif in *Xist* RepA 104. Consistently, several studies demonstrated that PRC2 showed high affinity to G-tract-containing RNA molecules and preferentially bound to G-quadruplex structures, but displayed much lower binding affinity to RNA duplexes 105 (Figure 1B). Importantly, PRC2's preferential binding to RNA G-quadruplex is evolutionarily conserved 106. Another oncogenic lncRNA, HERES, promotes Wnt signaling pathways through recruiting PRC2 to chromatin in esophageal squamous cell carcinoma and this recruitment is through direct binding of EZH2 to HERES 70. Interestingly, the GGW (W: A or U) repeats in HERES, which form the G-quadruplex-like motifs, are essential for its interaction with EZH2.

RNA G-quadruplexes are generally present in the 5'-region of the first intron 107, which may explain why nascent RNAs may act as *cis-*regulatory elements in gene activation through binding to EZH2 and antagonizing the repressive activity of PRC2 54. Consistently, a recent report by Beltran *et al*. verified preferential binding of PRC2 to G-tracts in nascent pre-mRNA and further revealed that RNA G-quadruplex could attract PRC2 to evict it from chromatin, leading to specific gene activation 108. In addition to nascent RNAs, other lncRNAs, such as LINC-PINT, HOTAIRM1 and PPP1R1B, could also exert *cis*- or *trans*-regulatory roles to activate gene expression through sequestering PRC2 109-111.

**Figure 2:**
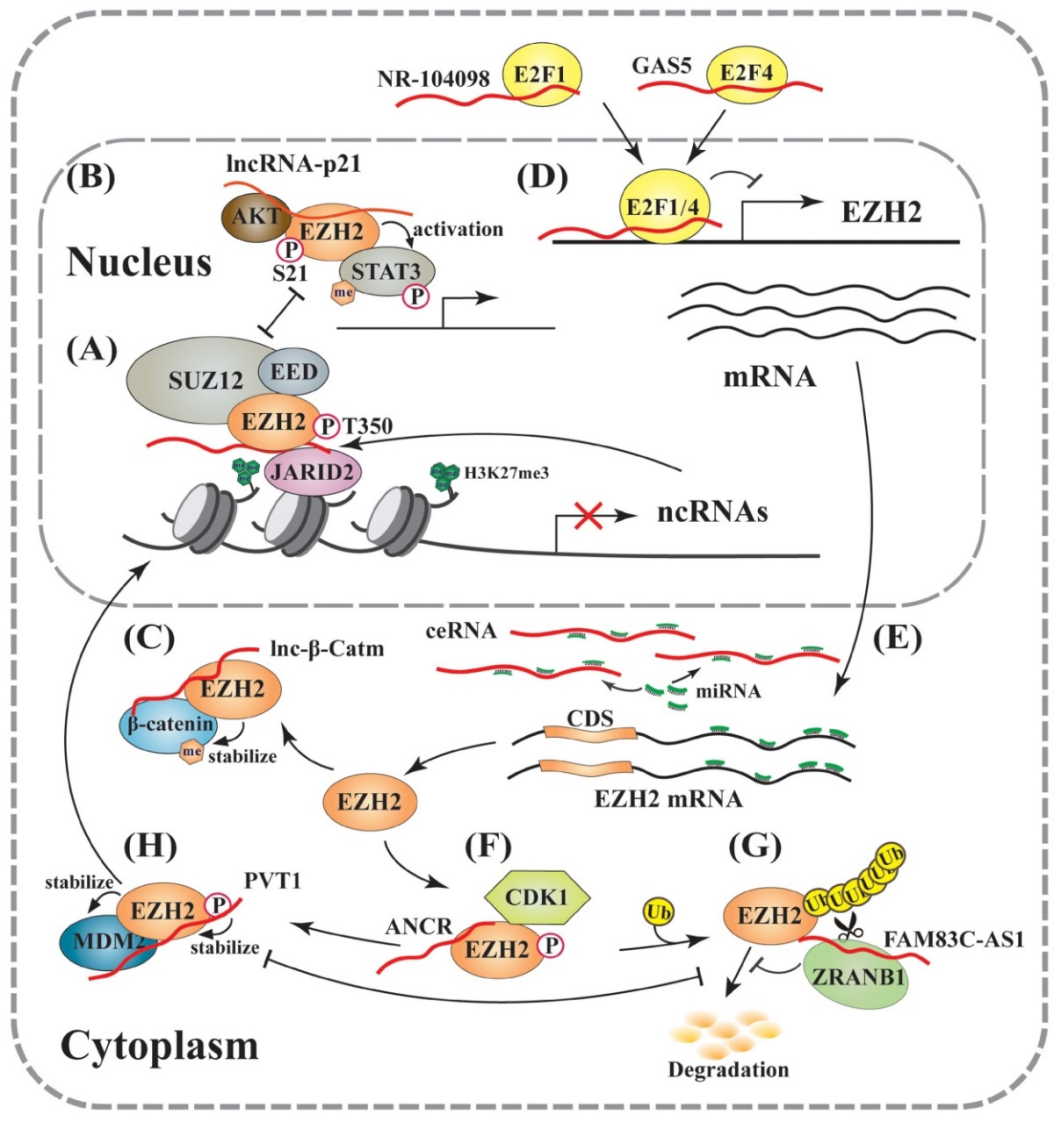
** LncRNAs involved in regulation of EZH2 expression and activity.** (A) The PRC2- or EZH2-associated lncRNAs regulate the expression of various genes, including lncRNAs. Many lncRNAs inhibit EZH2 methyltransferase activity, which can be relieved by JARID2 binding leading to reduced PRC2-RNA interaction and subsequently enhanced EZH2 activity. (B) EZH2 directly methylates non-histone proteins independent on PRC2: LincRNA-p21 disrupts PRC2 through competitively binding EZH2, and enhances the EZH2-pS21 by AKT, resulting in the coactivation of methylated STAT3. (C) Lnc-β-Catm stabilizes β-catenin protein through directly promoting its methylation by EZH2. (D) At the transcriptional level: LncRNAs, such as NR-104098 and GAS5, suppress EZH2 transcription through recruiting transcription factors, such as E2F1 and E2F4, respectively. (E) At the post-transcriptional level: ceRNAs scavenge microRNAs, and thus prevent the EZH2 mRNA from degradation and translational inhibition. (F), (G) and (H) At the post-translational level: lncRNAs, such as ANCR, directly bind EZH2 and enhance pT350 through increasing its interaction with CDK1, leading to EZH2 ubiquitination and proteasomal degradation (F). FAM83C-AS1 recruits deubiquitinase ZRANB1 to stabilize EZH2 (G). PVT1 improves EZH2 stability through blocking EZH2-pT350 to antagonize its ubiquitination, and EZH2 can also stabilize MDM2 through direct interaction. PVT1 also recruits EZH2 to repress gene transcription (H).

Thus, RNAs possess a dual role in regulating PRC2-mediated gene expression. In the *cis*-acting regulation, nascent RNAs can be scanned by PRC2, which allows it to bind RNA G-tracts or G-quadruplex motifs. The recruited PRC2 can enhance H3K27me3 in the promoter to strengthen transcriptional repression. However, if nascent RNAs are quickly transcribed, the pre-existing PRC2 on chromatin can bind G-rich RNAs and be promptly removed from the promoter, leading to reduced H3K27me3 on a targeted promoter and consequent gene activation. Therefore, the decision between recruitment and eviction of PRC2 on a target gene depends on the status of RNAs, including both nascent RNAs and lncRNAs 26, 47, 105 (Figure 1C). In a study of oncogene-induced senescence, Muniz *et al*. discovered that an isoform of the circular ANRIL could bind to Polycomb proteins and reduce EZH2 occupation on the p15 and p16 promoters, leading to declined H3K27me3. As a result, the expression of ANRIL or its relative levels versus EZH2 determined the expression of these genes, as well as cell senescence 112. Noteworthily, many lncRNAs are downstream effectors of EZH2, and over 20% of lncRNAs are regulated by PRC2 (Figure 2A) 113. Several lncRNAs acting as guide partners of PRC2, such as MEG3 and SPRY4-IT1, were also found to be suppressed by EZH2 114, 115.

To date, mechanisms of RNA-mediated *trans*-regulation for EZH2 eviction are still poorly understood, owing to the highly complex and dynamic features of epigenetic regulation. With the applications of RNA-immunoprecipitation followed by high-throughput sequencing (RIP-seq) technology, more PRC2/EZH2-associated lncRNAs have been identified and annotated, and their functional roles will be explored and expectantly revealed in further research 113, 116. In a report by Ye *et al*., the authors demonstrated that 2,595 previously annotated lncRNAs were potentially associated with EZH2 in neuroblastoma cells 113 In another study, Wang *et al*. carried out a global screening for EZH2-binding lncRNAs in different tissues, and identified 1,328 EZH2-associated lncRNAs. Among them, 470 lncRNAs were detected in at least two tissues, while 858 were only present in single tissues. Although a potential EZH2-binding motif was discovered in many lncRNAs, but it was not found in all immunoprecipitated RNAs 116. Thus, EZH2 is a protein with promiscuous binding affinity to different lncRNAs, and its detailed RNA binding domain(s) needs to be prudently mapped in future studies.

## LncRNAs involved in PRC2-independent methylation of non-histone proteins

As a methyltransferase, EZH2 has also been reported to directly promote the methylation of many non-histone proteins. For instance, EZH2-mediated K299 mono-methylation of GATA4 reduced its transcription factor activity through disrupting its binding to p300 and consequently decreasing its acetylation 117. In another report, Lee *et al*. discovered methylation-dependent ubiquitination machinery. Through this mechanism, non-histone proteins, such as RORα, were mono-methylated by EZH2, and the methylation status could be recognized by a specific E3 ligase to promote their ubiquitination 118, which is independent of EZH2's activity in mediating H3K27me3.

On the other hand, EZH2 could also manifest its oncogenic activity through acting as a coactivator to promote the methylation of transcription factors, such as androgen receptor in prostate cancer and STAT3 in glioblastoma 119, 120. AKT can mediate EZH2 phosphorylation at S21 (pS21). A number of reports demonstrated that pS21 of EZH2 reduced its activity in promoting H3K27me3 120-122, or negatively correlated with this gene silencing marker 119, 120. Consistently, inhibition of the PI3K-AKT pathway could decrease EZH2-pS21 but increase the global levels of H3K27me3 120, suggesting that pS21 acts as a molecular switch of EZH2 between a repressor and an activator. A recent study revealed that lincRNA-p21 could enhance EZH2-pS21 through inhibiting the EZH2-HOTAIR interaction, leading to PRC2 disruption in prostate cancer cells 123, indicating that lincRNA-p21 determines the outcomes of EZH2-mediated gene expression (Figure 2B). It supports a *trans*-regulatory mechanism of lncRNAs through competitively binding EZH2 to activate gene expression. In addition, β-catenin, the key effector of the Wnt signaling pathway and a signal transducer to the nucleus, was reported to be methylated at K49 by EZH2, which increased its stability through blocking ubiquitination. In this process, a lncRNA, lnc-β-Catm, could bind to both β-catenin and EZH2 to promote β-catenin methylation and improve its stability 124 (Figure 2C). Importantly, this regulatory activity of EZH2 is independent of PRC2, since depletion of SUZ12 and EED exerted no effect on it.

## LncRNAs involved in regulating EZH2 expression and activity

Accumulating evidence indicates that lncRNAs regulate the expression and activity of EZH2 at different levels, including transcription, post-transcription and post-translational modifications.

### LncRNA-mediated transcriptional regulation of EZH2

Early studies demonstrated that EZH2 expression was regulated by various transcription factors, including MYC, SOX4 and E2Fs 125-127. The E2F family is a group of DNA-binding proteins that play a critical role in promoting cell cycle progression 128. Whether an E2F protein activates or represses gene expression depends on its associated cofactors 129-132. Two recent studies indicated that both E2F1 and E2F7 could enhance EZH2 transcription through binding to its promoter 133, 134. Consistently, an E2F binding site was identified in the EZH2 promoter 135-137. However, Feng *et al*. reported that E2F1 repressed EZH2 expression when recruited by a lncRNA, NR-104098, to the EZH2 promoter 138 (Figure 2D). Based on this mechanism, treatment of acute myelogenous leukemia cells by ATPR, a derivative of all-trans retinoic acid (ATRA), could markedly stimulate NR-104098 expression and subsequently block EZH2 expression, leading to reduced cell proliferation and enhanced cell differentiation 138.

As a well-characterized lncRNA, GAS5 is downregulated in solid tumors, and its decreased expression correlated with poor prognosis of cancer patients 136, 139-141. Mechanistically, GAS5 could bind E2F4, recruit it to the EZH2 promoter, and repress EZH2 expression, leading to enhanced apoptosis and reduced viability of bladder cancer cells 136 (Figure 2D). Consistently, Xu *et al*. also reported that GAS5-mediated EZH2 inhibition could decrease H3K27me3 and consequently upregulate CDKN1C expression, causing accelerated oxidative stress and apoptosis of melanoma cells 142. All these findings extended our understanding of lncRNAs involved in EZH2 transcription, and suggested new therapeutic targets in cancer therapies.

### LncRNAs-mediated post-transcriptional regulation of EZH2

MicroRNAs are small endogenous ncRNAs regulating gene silencing through promoting destabilization and blocking translation of target mRNAs. MicroRNA expression profiles can be used to classify cancers, and the levels of specific microRNAs correlate with diagnosis and prognosis of cancer patients 143, 144. Many groups, including ours, reported that EZH2 expression was regulated by microRNAs, such as miR-101 and miR-26a 145, 146. It is also interesting to know that EZH2 can generally inhibit the expression of EZH2-targeting microRNAs through promoting the H3K27me3 of their genomic loci to create a positive feedback loop that maintains high EZH2 expression and malignant status 147. A microRNA can be specifically blocked by its antisense oligonucleotide, such as a locked nucleic acid (LNA) with its complementary sequence, or an RNA containing multiple copies of its binding site to act as a microRNA sponge, leading to reduced microRNA binding to the target mRNA and its upregulated gene expression 148, 149. In addition, this regulatory mechanism of microRNAs' action also exists through a concept of competing endogenous RNA (ceRNA) 150, 151 (Figure 2E).

The first reported microRNA targeting EZH2 is miR-26a, which is overexpressed during myogenesis 145, 152. Recently, the lncRNA, SNHG6, was determined as a ceRNA to trap miR-26a and consequently promote EZH2 expression in colorectal cancer 153. Importantly, SNHG6 has also been identified as a molecular sponge of miR-101, -214 and -4465, which all target EZH2 in different cancers 154-156. The term “molecular sponge” was designated as a relatively large ncRNA containing multiple binding sites of one or more microRNA molecules and thus sequestering them. In addition, a number of other lncRNAs could also work as decoys to antagonize different microRNAs and thus promote EZH2 expression, leading to enhanced cancer progression and resistance to chemo- or radiotherapies (Table 2). As we indicated above, many lncRNAs work with the EZH2 protein to mediate H3K27me3 at specific genomic loci. Therefore, lncRNAs can modulate the function of EZH2 at multiple levels.

### LncRNA-mediated post-translational modifications of EZH2

EZH2 activity and stability are regulated by different post-translational modifications, including phosphorylation, ubiquitination, methylation and O-GlcNAcylation 98, 121, 169-173, and many lncRNAs contribute to these processes. As discussed above, pT350 is crucial for EZH2 binding to lncRNAs and PRC2 recruitment to chromatin. Activities of the lncRNA ANCR in binding EZH2 and promoting its phosphorylation have been reported by several research groups, but the overall effects of this regulation on cancer cells varied, likely depending on cancer types and genetic backgrounds. Li *et al*. reported that ANCR could directly bind mouse Ezh2, promote its interaction with CDK1, and consequently increase pT345 and pT487, leading to enhanced Ezh2 ubiquitination and degradation, and reduced breast cancer progression 31 (Figure 2F). Consistently, in another report, ANCR inhibited proliferation, migration and invasion of osteosarcoma cells through binding and attenuating EZH2 174. However, a positive regulation of EZH2 expression by ANCR was also reported in colorectal cancer and glioma cells 59, 175, as well as osteoblast cells 176.

Several recent studies revealed the activities of additional lncRNAs in promoting EZH2 ubiquitination and degradation, including MEG3, UCA1 and the circular RNA circ-ADD3 177-179. Interestingly, all these lncRNAs exerted their regulation through promoting EZH2's interaction with CDK1 and enhancing its phosphorylation, which subsequently caused EZH2 ubiquitination and degradation. As for the regulatory mechanism, Li *et al*. proposed that lncRNA binding likely altered EZH2 conformation that improved recognition by CDK1 to facilitate its phosphorylation 25. Another lncRNA PAR5, downregulated in anaplastic thyroid carcinoma, was also reported to negatively regulate EZH2 activity. PAR5 interacts with EZH2, decreases its protein levels, and reduces its binding to the E-cadherin promoter 180. Consistently, similar regulation of EZH2 by PAR5 was also observed in human glioma 181.

On the other hand, lncRNA FAM83C-AS1 was shown to stabilize EZH2 protein through promoting its binding to ZRANB1, a deubiquitinase (Figure 2G). Upregulated EZH2 could mediate the H3K27me3 in the promoter of SEMA3F, which promoted colorectal cancer development 182. In addition, lncRNAs PVT1 and HERES were both reported to promote EZH2 protein stability with undetermined molecular mechanisms 70, 183. Based on the evidence of available studies, we predict that the *trans* recruitment of EZH2 by lncRNAs to chromatin can concurrently reduce its ubiquitination through blocking T350 phosphorylation, and consequently stabilizing EZH2.

Several lncRNAs have been reported to negatively regulate EZH2 stability or activity. EDAL is a lncRNA that inhibits the replication of neurotropic viruses in neuronal cells. The mechanism underlying this regulation is EDAL-blocked T309 O-GlcNAcylation of EZH2. Interestingly, although T309 is involved, EDAL did not apparently alter the phosphorylation of EZH2 32. As discussed above, lincRNA-p21 can dramatically enhance AKT and EZH2 interaction, which consequently leads to increased pS21 of EZH2, and promotes EZH2-mediated STAT3 methylation 123. Additionally, pS21 of EZH2 disrupts EZH2 binding to histone H3 and subsequently reduces H3K27me3 of target genes 121.

Actually, a positive feedback regulation exists between lincRNA-p21 and p53; while p53 transactivates lincRNA-p21 gene expression, the lncRNA also antagonizes MDM2-mediated p53 ubiquitination and degradation 184-187. In addition, EZH2 serves as a signal relay between lncRNA PVT1 and p53; PVT1 improves EZH2 protein stability, while EZH2 also physically interacts with and stabilizes MDM2 (Figure 2H), leading to enhanced p53 degradation 183, 188, 189. MEG3, which promotes EZH2 degradation 177, was also reported to downregulate MDM2 and subsequently activate the p53 pathway 190. On other hand, MDM2's physical interaction with EZH2 on chromatin promotes H3K27me3, which suppresses lineage-specific genes and favors efficient generation of induced pluripotent stem cells (iPSCs) 191.

Based on this complex regulatory network, EZH2 is causally relevant to p53 activation. Thus, these lncRNAs regulating EZH2 stability and activity can either directly modulate EZH2-related epigenetic modifications or indirectly alter p53 signaling pathways through a positive EZH2-MDM2 interplay.

## Conclusion and prospective

LncRNAs can regulate both the expression and methyltransferase activity of EZH2. In this review, we summarized this complex regulatory network in the following three aspects. First, during transcriptional initiation, lncRNAs and nascent RNAs can either recruit PRC2 to target promoters or evict it from chromatin, which determines the repressive or active expression status of a target gene, respectively (Table 1 and Figure 1C). Second, regarding target selection, lncRNAs regulate EZH2-mediated methylation in either histone or non-histone proteins, which can both determine target proteins' activities and alter global H3K27me3 levels (Figures 2A, 2B and 2C). Third, lncRNAs modulate EZH2 expression through directly regulating its gene transcription and indirectly acting as ceRNAs to scavenge EZH2-targeting microRNAs (Figures 2D, 2E and Table 2). Fourth, at the protein level, lncRNAs are also involved in the regulation of EZH2 post-translational modifications, which impacts its methyltransferase activity and also the activity or stability of its binding partners (Figures 2B, 2C, 2F-2H). Taken together, the complex regulation of EZH2 by lncRNAs at different levels just represents a tip of the iceberg in the whole ncRNA regulatory network. Deep understanding of additional mechanisms underlying lncRNA-mediated EZH2 actions, as well as other key cancer-related genes and proteins, will heavily rely on continuous exploration by scientific researchers and the advance of research technologies. Nevertheless, the current understanding of EZH2 expression and activity mediated by lncRNAs will not only be exemplificative in the studies of other oncogenic networks regulated by lncRNAs, but also provide insights in discovering novel therapeutic targets of cancer treatments.

## Figures and Tables

**Table 1:** LncRNAs recruiting EZH2 to inhibit gene transcription in cancers.

LncRNA	Expression	Cancer type	Interacting molecules	Cell process / Clinical feature
AGAP2-AS1	Up	GBM^57^	EZH2, LSD1	Proliferation, apoptosis, invasion / Shorter OS
ANCR	Up	BC*^58^, glioma^59^	EZH2	Proliferation, migration & invasion^58^, ^59^, apoptosis, EMT & stemness^59^ / Lymph node metastasis, advanced TNM stages^58^
AWPPH	Up	BC#^60^, NPC^61^	EZH2^60^, ^61^, LSD1^61^	Proliferation, apoptosis, migration^60^, ^61^, autophagy^60^ / -
BLACAT1	Up	PC^62^	EZH2	Proliferation, migration, AG and MOP / -
CASC9	Up	BC#^63^, ESCC^64^	EZH2	Proliferation^63^, ^64^, migration & invasion^63^, apoptosis^64^ / Larger tumor size, shorter OS^64^
FOXC2-AS1	Up	Melanoma^65^	EZH2	Proliferation, apoptosis / Metastasis, shorter OS
FOXD2-AS1	Up	HCC^66^	EZH2	Proliferation / Larger tumor size, shorter OS and DFS
FOXP4-AS1	Up	NSCLC^67^, osteosarcoma^68^	EZH2, LSD1^67^, ^68^	Proliferation, apoptosis, migration & invasion^67^, ^68^ / Larger tumor size, advanced TNM stage, shorter OS and PFS^67^
H19	Up	BC#^69^	EZH2	Metastasis / Invasion
HERES	Up	ESCC^70^	EZH2	Cell cycle, apoptosis / Worse survival
HOTAIR	Up	BC*^20^, ^71^-^73^, OSCC^74^, PC^75^	EZH2	Proliferation, apoptosis, migration & invasion^74^, ^75^, EMT & stemness, DNA damage repair, radio-resistance^20^, ^71^-^73^ / Metastasis and survival^20^, ^71^-^73^, advanced clinical stage^75^, lymph node metastasis and shorter survival^74^
HOXD-AS1	Up	GC^76^, osteosarcoma^77^	EZH2^76^, ^77^	Proliferation^77^, cisplatin resistance^76^ / Lymph node metastasis, advanced TNM stage^76^, shorter OS^76^, ^77^
LINC-PINT	Down	Melanoma^78^	EZH2	Proliferation, migration / Shorter OS
MAGI2-AS3	Down	Esophageal cancer 79	EZH2	Proliferation, apoptosis, radio-resistance / -
MALAT1	Up	RCC^80^	EZH2	Proliferation, apoptosis, invasion / Advanced clinical stage and shorter OS
MEG3	Down	BC*^47^, ^81^, ^82^	EZH2, JARID2	Proliferation, invasion, angiogenesis / -
PVT1	Up	GC^83^, NSCLC^84^, thyroid cancer^85^	EZH2	Proliferation 83-85, apoptosis 84 / Larger tumor size^84^, deeper invasion depth^83^, lymph node metastasis and advanced TNM stag, shorter survival^83^, ^84^
SNHG6	Up	Chondrosarcoma^86^, GC^87^	EZH2	Proliferation^86^, ^87^, migration & metastasis / Deeper invasion depth and advanced TNM stage^87^, clinical classification^86^
SNHG7	Up	OC^88^	EZH2	Growth, migration & invasion, metastasis, EMT / -
SPRY4-IT1	Up	CCA^89^	EZH2, LSD1, DNMT1	Growth, metastasis / Tumor node metastasis, worse OS and PFS
TUG1	Up	SCLC^90^	EZH2	Proliferation, migration & invasion, apoptosis, chemo-sensitivity / Worse OS
	Down	NSCLC^91^, ^92^	EZH2^91^, ^92^, EED^92^	Proliferation^91^, ^92^ / Larger tumor size and advanced pathological stage^91^
Xist	Up	NSCLC^93^, NB^94^	EZH2^93^, ^94^	Growth^93^, migration & invasion^94^, metastasis^93^ / larger tumor size and advanced TNM stage, shorter OS^93^

BC*: breast cancer; BC#: bladder cancer; CCA: cholangiocarcinoma; ESCC: esophageal squamous cell carcinoma; GBM: glioblastoma; GC: gastric cancer; HCC: hepatocellular carcinoma; NB: neuroblastoma; NPC: nasopharyngeal carcinoma; NSCLC: non-small cell lung cancer; OC: ovarian cancer; OSCC: oral squamous cell carcinoma; PC: pancreatic cancer; RCC: renal cancer Carcinoma; SCLC: small cell lung cancer; AG: aerobic glycolysis; MOP: mitochondrial oxidative phosphorylation; EMT: epithelial-mesenchymal transition; TNM: tumor node metastasis; OS: overall survival; DFS: disease-free survival; PFS: progression-free survival.

**Table 2:** CeRNAs scavenging microRNAs that target EZH2 mRNA.

CeRNA	Cancer type	MicroRNAs	Cellular activity	Clinical relevance
FOXC2-AS1	PCa^157^	miR-1253	Proliferation, tumor growth	Shorter survival
H19	NPC^158^	miR-630	Invasion	-
HOTAIR	OC^159^	miR-138-5p	Cisplatin resistance	-
MALAT1	OSCC^160^ GC^161^	miR-101^160^, miR-124-3p^161^	Proliferation^160^, ^161^, invasion^160^, migration, and H2 resistance^161^	-
NEAT1	EC^162^	miR-144-3p	Proliferation, migration & invasion	-
PVT1	NSCLC^163^	mi-526b	Proliferation, migration	Shorter survival
SNHG6	CRC^155^, ESCC^154^, OCCC^156^	miR-101-3p^154^, miR-214^155^, miR-26a/b^153^, ^155^	Apoptosis^154^, ^156^, migration & invasion, metastasis^153^, ^155^, ^156^, EMT^153^	Deeper invasion depth, lymph node metastasis and advanced TNM stage, shorter survival^155^, ^156^
SPRY4-IT1	BC#^89^, ^164^	miR-101-3p^89^, ^164^	Metastasis^89^, ^164^, proliferation, migration & invasion^164^	Tumor node metastasis, worse OS and PFS^89^
TUG1	PC^165^	miR-382	Proliferation, migration and EMT	Large tumor size, advanced TNM stage, shorter survival
Xist	CRC^166^, GC^167^	miR-137^166^, miR-101^167^	Proliferation^167^, migration & invasion^166^, ^167^	Large tumor size, lymph node invasion and advanced TNM stage, distant metastasis and worse OS^167^
ZNFX1-AS1	CRC^168^	miR-144-3p	Proliferation, migration & invasion, metastasis	Larger tumor size, deeper invasion depth, lymph node invasion, and advanced TNM stage, shorter survival

EC: endometrial cancer; OCCC: ovarian clear cell carcinoma; PCa: prostate cancer.

## Abbreviations

AGAP2-AS1: AGAP2 antisense RNA 1
AKT: serine/threonine kinase 1
ANCR: differentiation antagonizing non-protein coding RNA
BLACAT1: bladder Cancer associated transcript 1
CASC9: cancer susceptibility 9
CDK1/2: cyclin-dependent kinase ½
CDKN1C: cyclin dependent kinase inhibitor 1C
E2F: E2F transcription factor
EDAL: EZH2 degradation associated lncRNA
EED: Embryonic Ectoderm Development
FAM83C-AS1: FAM83C antisense RNA 1
FOXC2-AS1: FOXC2 antisense RNA 1
FOXD2-AS1: FOXD2 adjacent opposite strand RNA 1
FOXP4-AS1: FOXP4 antisense RNA 1RBBP4/7, RB binding protein 4/7
GAS5: growth arrest specific 5
GATA4: GATA binding protein 4
H19: H19 imprinted maternally expressed transcript
HERES: highly expressed lncRNA in esophageal squamous cell carcinoma
HOTAIRM1: HOXA transcript antisense RNA, myeloid-specific 1
HOXD-AS1: HOXD antisense growth-associated long noncoding RNA
JARID2: jumonji and AT-Rich interaction domain containing 2
lincRNA-p21: tumor protein p53 pathway corepressor 1
LINC-PINT: long intergenic non-protein coding RNA, p53 induced transcript
LSD1: lysine specific demethylase 1
MAGI2-AS3: MAGI2 antisense RNA 3
MALAT1: metastasis associated lung adenocarcinoma transcript 1
MEG3: maternally expressed 3
MYC: proto-oncogene, BHLH transcription factor
NEAT1: nuclear paraspeckle assembly transcript 1
PAR5: Prader Willi/Angelman region RNA 5
PPP1R1B: protein phosphatase 1 regulatory inhibitor subunit 1B
RBBP4/7: RB binding protein 4/7, chromatin remodeling factor
RORα: RAR related orphan receptor A
SEMA3F: semaphorin 3F
SNHG6: small nucleolar RNA host gene 6
SNHG7: small nucleolar RNA host gene 7
SOX4: SRY-box transcription factor 4
SPRY4-IT1: SPRY4 intronic transcript 1
STAT3: signal transducer and activator of transcription 3
TUG1: taurine up-regulated 1
UCA1: urothelial cancer associated 1
XIST: X inactive specific transcript
YY1: Yin Yang 1
ZNFX1-AS1: ZNFX1 antisense RNA 1
ZRANB1: zinc finger RANBP2-Type containing 1
